# Assessing corn recovery from early season nutrient stress under different soil moisture regimes

**DOI:** 10.3389/fpls.2024.1344022

**Published:** 2024-03-06

**Authors:** Solomon Amissah, Godfred Ankomah, Robert D. Lee, Calvin D. Perry, Bobby J. Washington, Wesley M. Porter, Simerjeet Virk, Corey J. Bryant, George Vellidis, Glendon H. Harris, Miguel Cabrera, Dorcas H. Franklin, Juan C. Diaz-Perez, Henry Y. Sintim

**Affiliations:** ^1^ Department of Crop and Soil Sciences, University of Georgia, Tifton, GA, United States; ^2^ C. M. Stripling Irrigation Research Park, University of Georgia, Camilla, GA, United States; ^3^ Delta Research and Extension Center, Mississippi State University, Stoneville, MS, United States; ^4^ Department of Crop and Soil Sciences, University of Georgia, Athens, GA, United States; ^5^ Department of Horticulture, University of Georgia, Tifton, GA, United States

**Keywords:** adaptive nutrient management, nutrient stress, nutrient dilution effects, soil moisture, residual soil nutrients, corn productivity

## Abstract

Corn (*Zea mays*) biomass accumulation and nutrient uptake by the six-leaf collar (V6) growth stage are low, and therefore, synchronizing nutrient supply with crop demand could potentially minimize nutrient loss and improve nutrient use efficiency. Knowledge of corn’s response to nutrient stress in the early growth stages could inform such nutrient management. Field studies were conducted to assess corn recovery from when no fertilizer application is made until the V6 growth stage, and thereafter, applying fertilizer rates as those in non-stressed conditions. The early season nutrient stress and non-stress conditions received the same amount of nutrients. As the availability of nutrients for plant uptake is largely dependent on soil moisture, corn recovery from the early season nutrient stress was assessed under different soil moisture regimes induced via irrigation scheduling at 50% and 80% field capacity under overhead and subsurface drip irrigation (SSDI) systems. Peanut (*Arachis hypogaea*) was the previous crop under all conditions, and the fields were under cereal rye (*Secale cereale*) cover crop prior to planting corn. At the V6 growth stage, the nutrient concentrations of the early season-stressed crops, except for copper, were above the minimum threshold of sufficiency ranges reported for corn. However, the crops showed poor growth, with biomass accumulation being reduced by over 50% compared to non-stressed crops. Also, the uptake of all nutrients was significantly lower under the early season nutrient stress conditions. The recovery of corn from the early season nutrient stress was low. Compared to non-stress conditions, the early season nutrient stress caused 1.58 Mg ha^-1^ to 3.4 Mg ha^-1^ yield reduction. The percent yield reduction under the SSDI system was 37.6-38.2% and that under the overhead irrigation system was 11.7-13%. The high yield reduction from the early season nutrient stress under the SSDI system was because of water stress conditions in the topsoil soil layer. The findings of the study suggest ample nutrient supply in the early season growth stage is critical for corn production, and thus, further studies are recommended to determine the optimum nutrient supply for corn at the initial growth stages.

## 1 Introduction

Optimum plant nutrition is required to sustain plant health and productivity, especially for a high-input crop such as corn (*Zea mays*). Application of fertilizer to meet plant nutritional needs is therefore very critical in regions with highly weathered soil conditions. Oxisols and Ultisols, for instance, are highly weathered soils commonly found in tropical and subtropical regions. They are characterized by low organic matter, strong acidity, and poor native fertility because of rapid mineralization rates, intense weathering of primary minerals, and leaching of essential base cations (Juo and Franzluebbers, 2003; Chesworth et al., 2008; Weil and Brady, 2017; Nunes et al., 2019). This makes fertilizer a major input cost in corn production in the region. Moreover, instability in the supply and prices of fertilizers observed in recent years poses a lot of concern (Singh and Tan, 2022; Amissah et al., 2023b). Several fertilizers exceeded record prices in 2008, which affected the profit margin for growers (Singh and Tan, 2022). It is therefore imperative to optimize nutrient management in corn production.

Adaptive nutrient management that synchronizes nutrient supply with crop demand could increase nutrient use efficiency and minimize nutrient losses through runoff, leaching, ammonia volatilization, and denitrification (Esfandbod et al., 2017; Shahzad et al., 2018, 2019a; Amissah et al., 2023a). Crop nutrient uptake is low at the initial stages of growth, increasing towards the reproductive stage (Bender et al., 2013; Ciampitti and Vyn, 2014). A study conducted at two locations in the state of Illinois in the United States showed that by the six-leaf collar (V6) growth stage, <15% of macro- and micronutrients had been taken up by corn when compared to the total uptake at maturity (Bender et al., 2013). Also, biomass accumulation by the V6 stage was <5%. In conventional nutrient management, almost all the fertilizer rates are applied before planting or at the initial stages of planting, except for nitrogen, which is usually split-applied. In the southeast United States, with characteristic high rainfall and temperatures, fertilizers applied at the initial stages of growth are susceptible to losses, especially when the vegetation cover is minimal. Knowledge of corn recovery to early season nutrient stress could be used to better synchronize nutrient application with crop demand.

As corn is a high-input crop, it requires substantial levels of nutrients and water to sustain productivity. Moreover, the amount of water present in the soil largely affects the solubility and availability of applied mineral nutrients to crops (Pan et al., 2011; Sintim et al., 2015, 2016; Kusi et al., 2021b). Thus, there is a vital relationship between plant nutrient uptake and the status of soil water (Misra and Tyler, 2000; Djaman et al., 2013). Higher nutrient uptake levels have mainly been observed under adequate or fully irrigated conditions, while lower nutrient uptake is typical under water-limiting conditions (Setiyono et al., 2010; Djaman et al., 2013; Faloye et al., 2019). For instance, Djaman and Irmak (2018) observed reduced nitrogen (N) uptake when corn was irrigated below 75% of fully-irrigated treatment, with the fully-irrigated treatment being irrigation scheduling at 60% of total available water. The authors observed similar results for phosphorus (P) (Djaman and Irmak, 2018). Also, Seiffert et al. (1995) observed reduced potassium (K) uptake in corn with decreasing soil water content. The root length and K influx were also reduced by about 50% under the low soil water conditions (Seiffert et al., 1995). The lower nutrient uptake under water stress is usually due to decreased nutrient transport by mass flow and diffusion (Seiffert et al., 1995; Buljovcic and Engels, 2001; Djaman and Irmak, 2018). Thus, nutrients that are preferentially taken up by the mass flow pathway tend to restrict plant growth the most under dry conditions.

After nutrient uptake, plants assimilate, translocate, or remobilize nutrients, which determines nutrient use efficiency (Masclaux-Daubresse et al., 2010; Sintim et al., 2015, 2016). Moisture stress conditions have a negative impact on transpiration rate and stomatal conductance, ultimately impacting photosynthesis and nutrient assimilation and translocation (Masclaux-Daubresse et al., 2010; Fahad et al., 2017). Plants under moisture stress also have poor growth due to impaired cell growth as a result of reduced turgor pressure (Hussain et al., 2008). Irrigation is therefore an important management practice to supplement crop water demand. Overhead irrigation is a widely used irrigation method, which supplies water over the top of the plant canopy or soil surface. High amounts of water can be lost under overhead irrigation systems via evaporation and surface runoff, especially during the early growth stages with little to no soil cover. There has been increased interest in subsurface drip irrigation (SSDI), which entails supplying water through drip tapes installed below the soil surface. The method effectively reduces water loss via evaporation and surface runoff (Colaizzi et al., 2004; Hassanli et al., 2009). The different modes of water supply in overhead and SSDI irrigation systems change the soil moisture dynamics, and thus, different amounts of water may be needed to maintain the soil moisture content at a similar level. For corn production, irrigation scheduling at 50% field capacity (FC-50) is recommended as the standard to maximize net economic returns (Kisekka et al., 2016). However, the crop may experience some moisture stress. A study observed a significant yield difference in corn irrigated at FC-50 and 75% field capacity, with the FC-50 resulting in lower yields (Kebede et al., 2014).

Also, corn is particularly sensitive to nutrient stress in the early season, causing it to transition through its developmental stages quickly (Silva and Uchida, 2000; Grant et al., 2001; Roth et al., 2023). Thus, starter fertilizer, especially via band placement at 5.08 cm below the soil and 5.08 cm to the side of plant rows, is often applied at planting to induce early-season plant growth. However, the use of starter fertilizer does not always translate into better yield, especially under warm soil conditions, medium to high soil nutrient test levels, or when legume is the previous crop (Hoeft, 2000; Mallarino, 2015). There is limited information on whether corn will recover fully from nutrient stress in the early season under non-moisture stress conditions. Thus, the objective of the study was to assess corn recovery from early-season nutrient stress under different soil moisture regimes.

## 2 Materials and methods

### 2.1 Experimental site

Field experiments were established in 2021 and 2022 at the University of Georgia Stripling Irrigation Research Park in Camilla, GA (31°16’45.86”N, 84°17’29.65” W). The soil at the experimental site was Lucy loamy sand, classified as Loamy, kaolinitic, thermic Arenic Kandiudults, with an average sand, silt, and clay content of 90.7%, 3.2%, and 6.1%, respectively, at the 0-15 cm depth. The climate at the experimental site is subtropical, having average annual maximum, mean, and minimum air temperatures of 26.0°C, 19.4°C, and 12.8°C, respectively, with an annual rainfall of 1,314 mm and 98 average rainy days (Georgia AEMN, 2023). The minimum, average, and maximum air temperatures were 10.3°C, 20°C, and 27.7°C, respectively, in 2021, and 9.10°C, 19.7°C, and 28.1°C, respectively, in 2022. Annual rainfall was 1,419 mm in 2021 and 1,100 mm in 2022. Total rainfall received during the corn growing season (between planting and harvest) was 791 mm in 2021 and 629 mm in 2022.

### 2.2 Experimental approach

The field experiment entailed two irrigation scheduling thresholds [FC-50 and 80% field capacity (FC-80)], and two nutrient stress conditions [early season nutrient stress (ESN-stress) and non-nutrient stress (NN-stress)]. The treatment factors were laid in a split-plot randomized complete block design with four replications, and the experiment was established under two separate fields, with one field being equipped with an overhead irrigation system and the other field being equipped with an SSDI system. The two fields were 50 m apart in 2021 and 200 m apart in 2022. The irrigation schedules were the main plot factors, while the nutrient stress levels were the subplot factors.

The NN-stress entailed periodic nutrient application to ensure plant tissue nutrient levels were within recommended sufficiency levels for corn (Baker et al., 2000). The ESN-stress entailed no nutrient application until the V6 growth stage, after which the plots received a similar nutrient application as the NN-stress. The nutrient rates not supplied to the ESN-stress at the early stage were provided between the V6 and V7 growth stages. Thus, both nutrient stress levels received the same nutrient rates. Granular sources of nutrients were used as pre-plant fertilizer sources, and they were applied with a drop spreader, whereas in-season nutrient applications were by use of liquid side-dress applicators and injection through irrigation systems. Nutrient rates and main nutrient sources applied are presented in **Table 1**. Corn growth stage identification followed the University of Georgia Extension guideline (Bryant, 2021).

**Table 1:** Total nutrient rates and main nutrient sources applied in 2021 and 2022.

Nutrients	Rates in 2021	Rates in 2022	Main nutrient sources
	kg ha^-1^	kg ha^-1^	
N	336	303	Urea; Urea ammonium nitrate solution
P_2_O_5_	252	121	Diammonium phosphate; Ammonium polyphosphate solution
K_2_O	280	280	Potassium chloride; Potassium nitrate
Mg	5.60	5.60	Magnesium oxy-sulfate; Magnesium nitrate solution
Ca	11.2	5.60	Calcium chloride; Calcium sulfate; Calcium nitrate solution
S	11.2	11.2	Potassium sulfate; Ammonium thiosulfate solution
B	0.56	1.12	Fertilizer borate derived from Ulexite; Borosol^®^ 10 solution
Zn	1.12	1.68	Zinc oxysulfate; Zinc nitrate solution
Mn	1.68	3.36	Manganese sucrate; Manganese nitrate solution
Fe	1.68	2.24	Iron sucrate; Iron nitrate solution
Cu	0.56	0.56	Copper sulfate; Copper nitrate solution
Mo	0.00	0.22	Sodium molybdate solution

The irrigation schedule was determined by utilizing Teros 12 moisture sensors (METER Group, Inc., Pullman, WA, USA) initially installed at (a) 20 cm deep and 15 cm to the side of the plant row, and (b) 30 cm deep and 25 cm to the side of plant row to monitor soil moisture dynamics. The sensors were connected to a Zentra datalogger which was used to wirelessly transmit hourly soil moisture data. However, the sensors could not detect low rainfall events well. Thus, additional sensors and data loggers were obtained and installed at 10 cm deep and 15 to the side of plant rows, except for the FC-80 treatment under the SSDI system in 2021 due to limited supply. The available water holding capacity of the soil was calculated as the difference in water content at -33 kPa and -1500 kPa (Klute, 1986). **Table 2** provides the amount of irrigation water supplied under the different irrigation scheduling and application methods.

**Table 2:** Irrigation water supplied (in mm) under the different irrigation scheduling and application methods in 2021 and 2022.

Irrigation	——– FC-50 ——–		——– FC-80 ——–	
	2021	2022	2021	2022
Overhead	172	240	226	446
SSDI	117	244	189	470

SSDI, Subsurface drip irrigation; FC-50, Irrigation triggered at 50% field capacity; FC-80, Irrigation triggered at 80% field capacity.

### 2.3 Experimental field management

A lateral irrigation unit was used for the overhead irrigation system and had variable rate irrigation application capabilities. The SSDI system was set up by installing Netafim Typhoon drip tapes (Netafim Irrigation, Inc., Fresno, CA, USA) in the middle of plant rows at 30 cm soil depth. The fields were under cereal rye (*Secale cereale*) cover crop, and peanut (*Arachis hypogaea*) was the previous cash crop in both years. The cover crop was terminated by spraying Glyphosate [N-(phosphonomethyl) glycine] at the manufacturer-recommended rates, and the fields were prepared by strip-tilling to a depth of 30.5–45.7 cm before planting in March each year. The plot size was 12.2 m long by 5.49 m wide under the SSDI and 12.8 m long by 7.32 m wide under the overhead irrigation. Corn hybrid A6499STX by AgriGold was planted at the seeding rate of 88,958 seeds ha^-1^ and row spacing of 91.4 cm. Besides study treatments, standard agronomic and pest management recommendations by the University of Georgia Cooperative Extension were followed throughout the season to manage the experimental sites (Bryant, 2021).

### 2.4 Data collection

Initial nutrient levels of the soil were determined by sampling soils at 0-15 cm depth and sending them to the Waters Agricultural Laboratories, Inc. in Camilla, GA for analyses following standard procedures. Nitrate-N was measured with the automated flow injection analysis system (FIAlyzer-1000, FIAlab Instruments, Inc., Seattle, WA, USA) after extraction in a 2 M KCl solution. Extractable P, K, calcium (Ca), magnesium (Mg), iron (Fe), manganese (Mn), zinc (Zn), boron (B), and copper (Cu) were measured with an inductively coupled plasma optical emission spectrophotometer (ICP-OES; iCAP™ 6000 Series, Thermo Fisher Scientific, Cambridge, United Kingdom) after extraction with Mehlich I solution, and sulfur (S) was measured after extraction with monocalcium phosphate.

Before nutrient application in the ESN-stress plots, aboveground tissue samples were collected at the V6 growth stage within a uniform 1-m long strip of every plot and oven-dried, with oven set to 78°C until constant weight, to determine plant biomass. Nutrient analyses of the biomass samples were performed at the H.SINTIM LAB of the University of Georgia campus in Tifton, GA, following standard procedures. A 2400 Series II CHNS/O Elemental Analyzer (PerkinElmer U.S. LLC, Shelton, CT, USA) was used to measure the total N. Also, Avio 200 ICP-OES (PerkinElmer U.S. LLC, Shelton, CT, USA) was used to measure total P, K, Ca, Mg, S, Fe, Mn, Zn, B, Cu, and molybdenum (Mo) after sample digestion in nitric acid and hydrogen peroxide mixture using DigiPREP MS digestion block (SCP Science, Montreal, QC, Canada). Plant height (measured from the soil surface to the tallest leaf); ear height (measured from the soil surface to the base of the ear); ear length (length of the cob); ear diameter (measured from the center of the cob with grain intact); and ear grain rows (number of rows of corn grains on the cobs); and thousand seed weight (TSW) were determined at physiological maturity from plants within a uniform 1-m long strip. Also, the plants were partitioned into seeds and stover and dried in an oven to constant weight, and the weights were used to calculate the harvest index. The entire length of two rows of every plot was harvested with a plot combine harvester to obtain the seed weight and moisture content. The grain yield was determined at 155 g kg^−1^ moisture content.

### 2.5 Statistical analyses

The collected data were analyzed using a linear mixed model with the ‘lme4’ package in R (Bates et al., 2015). Separate statistical analyses were performed for the studies under the overhead irrigation and SSDI systems. The irrigation scheduling and nutrient stress factors were considered fixed effects, and year and block were considered random effects. Homoscedasticity of variance and assumptions of normality of residuals were assessed, and where appropriate, data transformation was performed using the Box-Cox transformation or the square root transformation methods. Separation of means was performed using the least square means and adjusted Tukey multiple comparison procedures with the ‘emmeans’ package in R (Lenth, 2018), and the significance level of all analyses was assessed at *P* = 0.05. The actual means and standard error of the data are reported in the tables and figures, which were followed by the mean separation letters obtained from the analyses. This approach avoids the need to back-transform data which sometimes produces values that are not consistent with the actual data.

## 3 Results and discussion

### 3.1 Initial soil nutrients

Initial nutrient concentrations of the soil are presented in **Table 3**. The initial NO_3_-N levels were generally low despite peanut, a legume, being the previous crop. However, the initial levels of the other nutrients were above the low threshold of the soil test classification for corn by the University of Georgia Extension, except for Zn in 2021 in the SSDI field (UGA-AESL, 2023). Nitrogen is usually not included in routine soil tests in the region because available forms of nitrogen do not accumulate (Kissel and Sonon, 2008; Hurisso et al., 2018). The sandy nature and high rainfall conditions in the region cause the available forms of nitrogen to readily leach, which could explain why low initial NO_3_-N levels were observed. Moreover, the use of rye cover crops could have depleted the soil of NO_3_-N. In addition, soils were sampled in late February, when temperatures were still low; thus, the peanut crop residuals may have not been sufficiently mineralized by the time of sampling (Grzyb et al., 2020; Shahzad et al., 2022; Amissah et al., 2023b). Mineralization of crop residues depends on several abiotic and biotic factors, including temperature, rainfall, soil properties, the chemical composition of crop residues, and the structure and composition of microbial communities (Whalen, 2014; Shahzad et al., 2019b; Grzyb et al., 2020; Sintim et al., 2022a; b).

**Table 3:** Initial nutrient status of the overhead and subsurface drip irrigation (SSDI) experimental field soil at 0-15 cm depth in 2021 and 2022.

Year	NO_3_-N	P	K	Mg	Ca	S	B	Zn	Mn	Fe	Cu
	———————————— Overhead (kg ha^-1^) ————————————										
2021	2.04	81.5	152	103	980	9.20	0.60	5.30	40.1	28.0	0.80
2022	1.96	81.5	152	103	979	9.25	0.62	5.30	40.1	28.0	0.78
	————————————– SSDI (kg ha^-1^) —————————————										
2021	1.60	38.1	76.8	62.7	800	10.1	0.28	1.35	19.6	10.1	4.99
2022	0.78	119	106	121	1131	24.6	0.31	6.11	43.1	26.1	1.12

Soil NO_3_-N was measured after extraction with 2 M KCl solution; soil P, K, Ca, Mg, Fe, Mn, Zn, B, and Cu were measured after Mehlich I extraction; and soil S was measured after extraction with monocalcium phosphate.

### 3.2 Soil water dynamics

**Figure 1:**
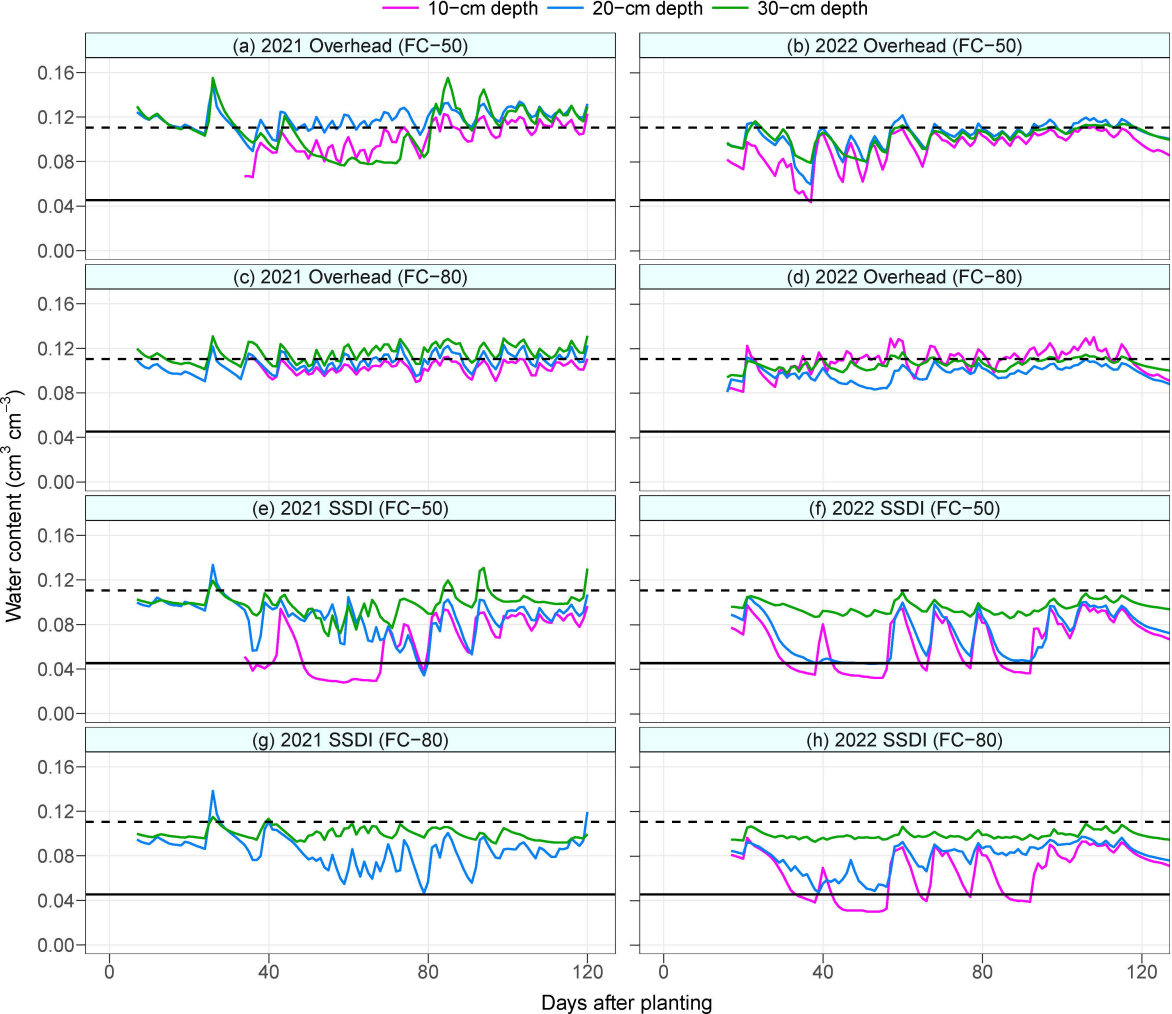
Mean daily soil water content at 10-cm, 20-cm, and 30-cm depths in 2021 **(A**, **C**, **E**, **G)** and 2022 **(B**, **D**, **F**, **H)**. Black solid and dashed horizontal lines indicate the permanent wilting point and field capacity of the soil, respectively. SSDI, Subsurface drip irrigation; FC-50, Irrigation triggered at 50% field capacity; FC-80, Irrigation triggered at 80% field capacity.

Soil moisture recharge at the 30-cm depth of the overhead irrigation at FC-50 was low during the mid-season in 2021. This triggered more irrigation under the overhead irrigation in 2021. The difference in irrigation water amount between the FC-50 and FC-80 of the overhead system was 54 mm in 2021 and 206 mm in 2022. Under the SSDI system, when soil moisture values of the sensors installed at 10 cm depth in 2021 and at 10 cm and 20 cm depths in 2022 are compared with those installed at the 30 cm depth, it can be seen that the sensors at the shallower depths could not detect irrigation events (**Figure 1**). The sensors were, however, able to detect rainfall events, demonstrating that the sensors were functional. As a result, the topsoil layer of the SSDI field was mostly below the permanent wilting point during the growing season, regardless of the irrigation scheduling threshold. In contrast, water content at the topsoil layer of the overhead irrigation was high, except for a single occasion when the water content under the FC-50 of the overhead irrigation went below the permanent wilting point in 2022. This was due to a technical constraint that delayed irrigation at the experiment station.

The inability of the soil moisture sensors at the topsoil layer to detect irrigation events under the SSDI system could be attributed to the configuration of the soil profile. The field has a top sandy layer underlaid by kaolinite clay minerals in the subsoil. Clay minerals have a larger surface area and more negative matric potential than sand (Jury and Horton, 2004). The drip tapes were installed at a 30-cm depth, which was at the interface of the sandy and clayey soil layer. Thus, the irrigation water would preferentially move downwards due to the higher capillarity of the clay layer and the downward exertion of the force of gravity (Abu-Zeid and El-Aal, 2017; Kroes et al., 2018; Rambabu et al., 2023). Irrigation water will only redistribute upwards to maintain equilibrium under very wet subsoil conditions.

### 3.3 Tissue nutrient concentration at the vegetative stage

The *P*-values of the main effects and interaction effects of irrigation schedule and nutrient stress on the concentration of plant nutrients at the vegetative stage are presented in **Supplementary Table S1**. The effects of the irrigation schedule on nutrient concentration were significant for only N under both the overhead and SSDI. Averaged across the fertility treatments under both irrigation scheduling methods, the FC-80 had a higher nutrient concentration, with a 6.07% and 7.65% increase in nutrient concentration under the overhead and SSDI, respectively, compared to the FC-50 (**Table 4**). The effects of nutrient stress on nutrient concentration were significant for N, P, Mg, Ca, Zn, Mn, and Mo under the overhead irrigation and for N, Ca, B, Zn, Mn, Cu, Fe, and Mo under the SSDI. The ESN-stress increased the concentrations of Mg, Ca, and Mo by 14.0%, 13.1%, and 30.5%, respectively, under the overhead irrigation and the concentrations of Ca, B, and Mo by 15.6%, 14.0%, and 63.3%, respectively, under the SSDI. In contrast, the NN-stress increased the concentrations of N, P, Zn, and Mn by 26.4%, 22.3%, 28.7%, and 100%, respectively, under the overhead irrigation and N, Zn, Mn, Cu, and Fe by 17.2%, 20.6%, 35.7%, 55.7%, and 20.7%, respectively, under the SSDI (**Table 4**). The interaction between irrigation schedule and nutrient stress was significant for only N under the overhead irrigation method, with the reduction in N concentration from ESN-stress being more severe under the FC-50 than under the FC-80 (24.3% vs. 17.4%). Similar observations were made under the SSDI (17.7% vs. 12.0%), except the interaction term was not significant.

**Table 4:** Effects of irrigation schedule and nutrient stress on nutrient concentrations of corn biomass sampled at the vegetative stage under overhead irrigation and SSDI systems.

Irrigation schedule	Nutrient stress	N	P	K	Mg	Ca	S	B	Zn	Mn	Fe	Cu	Mo
		————————— g kg^-1^ ————————————						————————– mg kg^-1^ ———————————–					
—————————————————————————————————— Overhead ———————————————————————————————													
FC-50	ESN-stress	34.6 ± 1.3a	4.00 ± 0.12ab	55.61 ± 0.93a	2.38 ± 0.12b	6.65 ± 0.24a	2.28 ± 0.19a	11.1 ± 0.4a	25.9 ± 1.7a	75.4 ± 5.2a	81.9 ± 10.3a	5.18 ± 0.48a	2.91 ± 0.41b
FC-50	NN-stress	45.7 ± 1.6c	4.63 ± 0.32bc	56.15 ± 1.26a	2.05 ± 0.12a	5.85 ± 0.13ab	2.36 ± 0.18a	10.8 ± 0.4a	32.1 ± 2.8b	151.4 ± 31.5b	89.8 ± 16.3a	5.68 ± 0.13a	2.09 ± 0.43a
FC-80	ESN-stress	38.5 ± 2.2b	3.79 ± 0.08a	55.85 ± 1.42a	2.34 ± 0.11ab	6.50 ± 0.20a	2.27 ± 0.16a	11.3 ± 0.3a	24.1 ± 2.0a	73.0 ± 4.1a	78.2 ± 16.1a	4.90 ± 0.38a	2.63 ± 0.42ab
FC-80	NN-stress	46.6 ± 1.8c	4.89 ± 0.29c	57.10 ± 1.55a	2.09 ± 0.18ab	5.77 ± 0.31b	2.32 ± 0.18a	10.7 ± 0.5a	32.3 ± 2.6b	145.5 ± 35.1b	87.7 ± 15.6a	5.56 ± 0.26a	2.15 ± 0.39a
——————————————————————————————————— SSDI ————————————————————————————————													
FC-50	ESN-stress	34.9 ± 2.2a	4.45 ± 0.18a	47.7 ± 2.1b	2.58 ± 0.23a	6.65 ± 0.56b	2.11 ± 0.13a	17.2 ± 0.7c	28.1 ± 4.6a	102.2 ± 6.4a	75.5 ± 9.6a	5.42 ± 0.72ab	2.87 ± 0.24b
FC-50	NN-stress	42.4 ± 1.0bc	4.75 ± 0.09a	49.1 ± 1.3a	2.49 ± 0.20a	5.58 ± 0.25a	2.18 ± 0.14a	14.6 ± 0.7a	31.7 ± 4.8b	151.5 ± 24.9b	91.9 ± 7.6b	6.72 ± 1.08ab	1.86 ± 0.26a
FC-80	ESN-stress	39.0 ± 0.9ab	4.40 ± 0.18a	46.0 ± 2.0b	2.61 ± 0.22a	6.44 ± 0.55b	2.22 ± 0.19a	16.2 ± 0.6bc	26.7 ± 3.6a	93.1 ± 12.6a	78.0 ± 8.1a	4.93 ± 0.33a	3.04 ± 0.26b
FC-80	NN-stress	44.3 ± 1.7c	4.55 ± 0.20a	48.9 ± 2.1a	2.40 ± 0.17a	5.74 ± 0.30a	2.36 ± 0.19a	14.8 ± 0.4ab	34.3 ± 6.5b	152.7 ± 32.7b	93.3 ± 8.2b	7.33 ± 1.14b	1.76 ± 0.29a

Within the irrigation application method (overhead or SSDI) and nutrient element, means not sharing any letter are significantly different using the least squares means and adjusted Tukey multiple comparisons (P < 0.05). Values represent the mean ± standard error. SSDI, Subsurface drip irrigation; FC-50, Irrigation triggered at 50% field capacity; FC-80, Irrigation triggered at 80% field capacity; ESN-stress, early season nutrient stress; NN-stress, reduced nutrient stress.

The greater reduction in N concentration from ESN-stress under the FC-50 could suggest water content at 50% of the soil’s field capacity was not sufficient to solubilize high amount of N. Water stress can drastically reduce the concentration of N since the amount of nutrients a plant can take depends on the volume of water available (Saud et al., 2017; Plett et al., 2020). The ESN-stress had almost all the plant tissue nutrient concentrations above the low threshold of the reference nutrient sufficiency ranges (NSR) reported in the Southern Cooperative Series Bulletin (SCSB) #394 (Baker et al., 2000), a publication of a regional collaboration that provides nutrient sufficiency ranges (NSRs) for plant analyses in the southern region of the United States (Baker et al., 2000). Only Cu, under the ESN-stress of the FC-80 for both overhead irrigation and SSDI, had concentrations below the NSR reported in the SCSB publication.

Nutrient concentration is affected by biomass accumulation, and hence, crops with good growth could have lower nutrient concentrations than those with poor growth, even though the actual nutrient uptake may be greater (Plénet and Lemaire, 1999; Adee et al., 2016; Rosa et al., 2019). The effect is commonly termed as ‘nutrient dilution effects.’ Therefore, nutrient concentration alone is not an adequate way to compare nutrient availability to crops. Research work by Amissah et al. (2023a) to assess the nutrient sufficiency ranges of corn in the SCSB publication showed some plots had more than 95% relative biomass at the V6-V7 growth stage even though the Cu levels were below the lower threshold. The findings highlighted that corn can tolerate lower Cu levels than reported. Moreover, Amissah et al. (2023a) observed that 25.4% of samples with all nutrient concentrations above the lower threshold even had relative biomass <50% and the effects were not due to the accumulation of nutrients at toxic levels. Thus, the thresholds of some nutrients can be higher or lower than what is currently reported, especially with the breeding of new corn varieties that are high-yielding and expected to require more nutrients (Bender et al., 2013).

### 3.4 Biomass and nutrient uptake at the vegetative stage

The *P*-values of the main effects and interaction effects of irrigation schedule and nutrient stress on plant biomass and nutrient uptake at the vegetative stage are presented in **Supplementary Table S2**. The effects of the nutrient stress on biomass were significant for both the overhead irrigation and the SSDI. The effects of the irrigation schedule and the interaction effects between the irrigation schedule and nutrient stress were not significant. Averaged over the irrigation schedule, the biomass of NN-stress was 2× greater than the biomass of ESN-stress under the overhead irrigation and 2.6× greater under the SSDI (**Table 5**). **Supplementary Figure S1** is a drone image of the SSDI field showing poor growth in the ESN-stress plots. The more severe effects of ESN-stress under the SSDI were likely because of the dry conditions experienced at the topsoil layer. Water regulates the transport of nutrients to the plant (Smethurst, 2004; Plett et al., 2020). Water stress will, therefore, lead to reduced uptake of water and nutrients and subsequently impact stomatal opening and absorption of carbon dioxide for photosynthesis. The effect will reduce growth, resulting in reduced biomass accumulation and partitioning (Chatzistathis and Therios, 2013; Bárzana and Carvajal, 2020; Pandey et al., 2021; Yan et al., 2023).

**Table 5:** Effects of irrigation schedule and nutrient stress on corn biomass and nutrient uptake at the vegetative stage under overhead irrigation and SSDI systems.

Irrigation schedule	Nutrient stress	Biomass	N	P	K	Mg	Ca	S	B	Zn	Mn	Fe	Cu	Mo
		———————————– kg ha^-1^ ———————————–							——————————– g ha^-1^ ——————————–					
——————————————————————————————– Overhead ———————————————————————————–														
FC-50	ESN-stress	214 ± 25a	7.55 ± 1.1a	0.85 ± 0.10a	11.9 ± 1.4a	0.52 ± 0.08ab	1.39 ± 0.13a	0.46 ± 0.03a	2.38 ± 0.29a	5.34 ± 0.54a	15.6 ± 1.4a	16.0 ± 1.4a	1.14 ± 0.20a	0.68 ± 0.16ab
FC-50	NN-stress	483 ± 38b	21.8 ± 1.3c	2.29 ± 0.29b	27.0 ± 1.9c	0.98 ± 0.07c	2.81 ± 0.19c	1.16 ± 0.16b	5.22 ± 0.44b	15.9 ± 2.23b	78.6 ± 19.5b	45.1 ± 9.5b	2.77 ± 0.26b	0.93 ± 0.14b
FC-80	ESN-stress	197 ± 20a	7.71 ± 1.0a	0.75 ± 0.08a	10.9 ± 1.1a	0.42 ± 0.09a	1.29 ± 0.15a	0.43 ± 0.04a	2.24 ± 0.26a	4.54 ± 0.35a	14.0 ± 1.2a	14.3 ± 2.7a	0.95 ± 0.11a	0.55 ± 0.13a
FC-80	NN-stress	356 ± 22b	14.5 ± 2.3b	1.76 ± 0.17b	20.3 ± 1.4b	0.74 ± 0.08bc	2.05 ± 0.18b	0.82 ± 0.08b	3.80 ± 0.30b	11.5 ± 1.12b	46.4 ± 13.5ab	31.3 ± 6.0ab	1.98 ± 0.17b	0.76 ± 0.14ab
———————————————————————————————————— SSDI ————————————————————————————————–														
FC-50	ESN-stress	176 ± 31a	6.09 ± 1.4a	0.79 ± 0.15a	8.69 ± 1.71a	0.41 ± 0.05a	1.11 ± 0.17a	0.35 ± 0.05a	2.92 ± 0.43a	4.21 ± 0.48a	17.8 ± 2.86a	13.8 ± 3.3a	0.86 ± 0.10a	0.53 ± 0.12ab
FC-50	NN-stress	443 ± 41b	15.3 ± 2.5b	2.10 ± 0.19b	21.58 ± 1.87b	1.15 ± 0.19b	2.52 ± 0.31b	1.00 ± 0.15b	6.57 ± 0.81b	15.08 ± 3.24b	71.9 ± 16.33b	40.2 ± 4.8b	3.22 ± 0.76b	0.79 ± 0.10b
FC-80	ESN-stress	153 ± 20a	5.52 ± 1.1a	0.66 ± 0.07a	7.25 ± 1.14a	0.38 ± 0.03a	0.94 ± 0.10a	0.32 ± 0.03a	2.43 ± 0.26a	3.64 ± 0.19a	14.4 ± 2.38a	12.9 ± 2.5a	0.73 ± 0.08a	0.48 ± 0.09a
FC-80	NN-stress	417 ± 45b	15.0 ± 2.6b	1.94 ± 0.28b	20.05 ± 1.86b	1.03 ± 0.16b	2.46 ± 0.38b	1.04 ± 0.18b	6.25 ± 0.80b	16.2 ± 4.32b	70.4 ± 19.63b	38.8 ± 5.7b	3.38 ± 0.78b	0.66 ± 0.07ab

Within the irrigation application method (overhead or SSDI) and nutrient element, means not sharing any letter are significantly different using the least squares means and adjusted Tukey multiple comparisons (P < 0.05). Values represent the mean ± standard error. SSDI, Subsurface drip irrigation; FC-50, Irrigation triggered at 50% field capacity; FC-80, Irrigation triggered at 80% field capacity; ESN-stress, early season nutrient stress; NN-stress, reduced nutrient stress.

In contrast to the results observed for nutrient concentration at the vegetative stage, the effects of nutrient stress on nutrient uptake were significant for all nutrients under both the overhead irrigation and the SSDI (**Table 5**). Under both irrigation scheduling methods, the ESN-stress caused a reduction in nutrient uptake. Irrigation scheduling significantly impacted the uptake of N, K, Mg, and Ca under overhead irrigation but not under the SSDI (**Table 5**). The interaction of irrigation scheduling and nutrient stress did not significantly impact nutrient uptake at the vegetative stage. Under the overhead irrigation, the ESN-stress caused a general reduction in nutrients. The nutrient uptake for the FC-50 irrigation scheduling method was higher than the FC-80, with the differences being significant for the N, K, Mg, and Ca under the overhead irrigation system. A similar trend was observed under the SSDI except for the uptake of S, Zn, and Cu where they were rather higher under the FC-80.

Reduction in nutrient uptake for the ESN-stress suggests the residual nutrients in the soil and mineralization of the peanut crop residues, which was the previous cash crop, could not supply adequate nutrients to corn by the V6 growth stage. Peanut is a leguminous crop that fixes atmospheric nitrogen, and it typically has 46 kg ha^-1^ to 80 kg ha^-1^ nitrogen in the aboveground biomass (Meso et al., 2007; Mulvaney et al., 2017). Moreover, peanuts are a good scavenger of residual nutrients in the soil, and thus, the decomposition of peanut residues can be a good source of nutrients for succeeding crops (Crusciol et al., 2021; Jat et al., 2023). The availability of nutrients from peanut residues to succeeding crops, is however, dependent on several management and environmental factors, including tillage operations, planting time, temperature, rainfall, soil properties, and the structure and composition of microbial communities (Whalen, 2014; Shahzad et al., 2019b; Grzyb et al., 2020; Sintim et al., 2022a; b).

Nutrient uptake of corn by the V6 growth stage was reported to be <15% of the total taken up by maturity (Karlen et al., 1988; Bender et al., 2013; Ciampitti and Vyn, 2013; Ciampitti et al., 2013). Despite the reduced nutrient uptake of corn in the early season, the results of the study demonstrate that nutrient supply must be optimum to sustain plant health and growth. Stress at any of the developmental stages of corn can affect biomass accumulation (Bender et al., 2013; Ciampitti et al., 2013). In contrast to corn, early-season nutrient stress (no nutrient application from planting to the square stage) was found to have no adverse effects on cotton growth and yield (Amissah et al., 2023b). Unlike corn, cotton is an indeterminate crop and can exhibit a high degree of plasticity in growth (Atkin et al., 2006; Rochester et al., 2012; Li et al., 2020; Kusi et al., 2021a; Amissah et al., 2023b).

### 3.5 Yield and growth parameters at maturity

**Figure 2:**
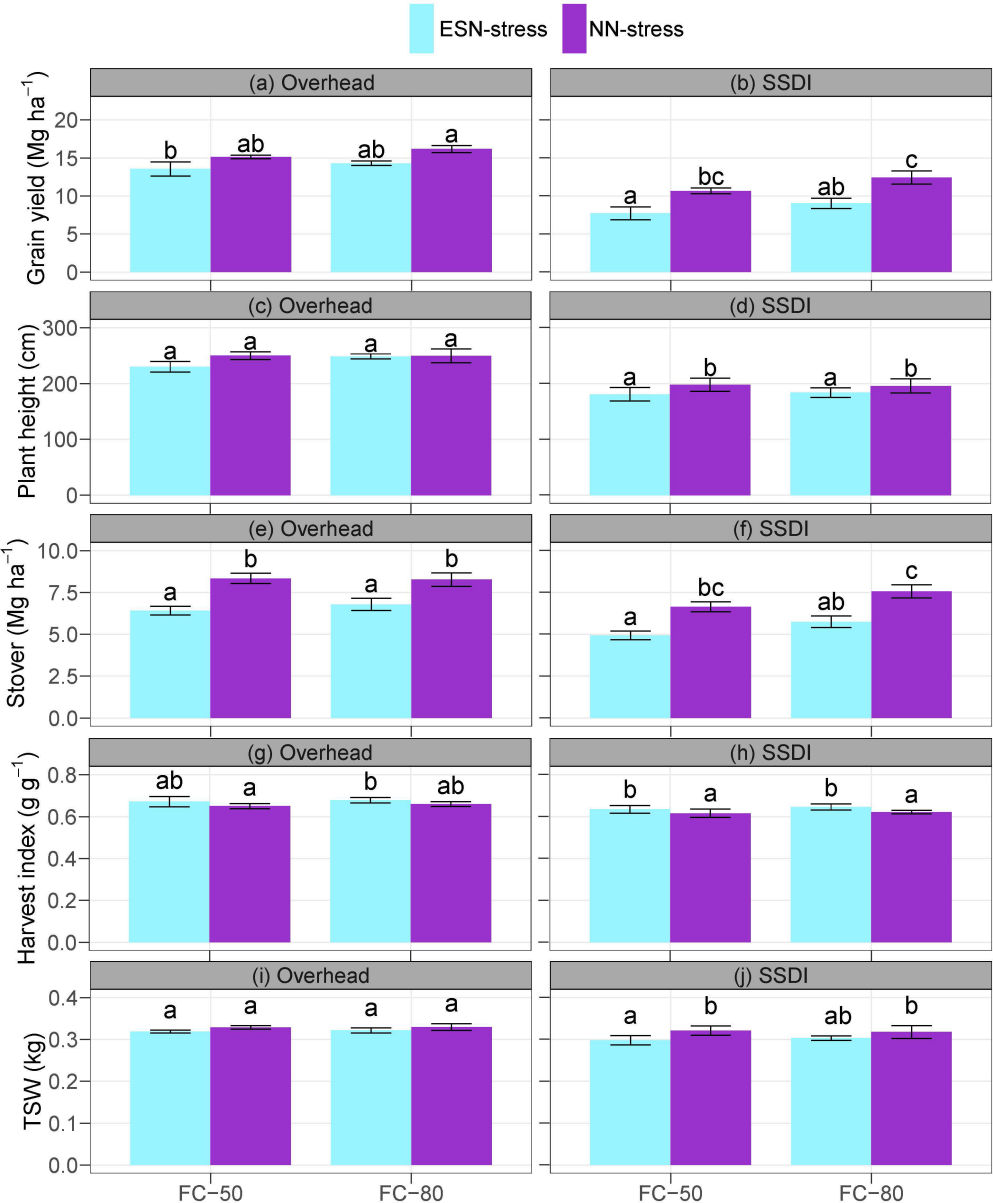
Effects of irrigation schedule and nutrient stress on corn yield **(A, B)** and growth parameters **(C–J)** at maturity under overhead irrigation and SSDI systems. Within the irrigation application method (overhead or SSDI) and measurement variable, bar plots of means not sharing any letter are significantly different using the least squares means and adjusted Tukey multiple comparisons (*P* < 0.05). Error bars indicate standard errors of the mean (n = 4). SSDI, Subsurface drip irrigation; FC-50, Irrigation triggered at 50% field capacity; FC-80, Irrigation triggered at 80% field capacity; ESN-stress, early season nutrient stress; NN-stress, reduced nutrient stress; TSW, Thousand seed weight.

The interaction effects of irrigation schedule and nutrient stress were not significant on grain yield under both the overhead irrigation and SSDI systems (**Supplementary Table S3**). The main effects of the irrigation schedule on grain yield were significant under just the SSDI system, but the main effects of nutrient stress on grain yield were significant under both the overhead and SSDI systems. Compared to the NN-stress, the ESN-stress caused a yield reduction of 1.58 Mg ha^-1^ (11.7%) and 1.86 Mg ha^-1^ (13.0%), respectively, for the FC-50 and FC-80 under the overhead irrigation system, and 2.95 Mg ha^-1^ (38.2%) and 3.4 Mg ha^-1^ (37.6%), respectively, for the FC-50 and FC-80 under the SSDI when compared to the NN-stress (**Figures 2A, B**). Under the SSDI, the FC-50 caused a 3.07 Mg ha^-1^ (16.7%) reduction in grain yield compared to the FC-80. Also, under the overhead irrigation, the FC-50 caused a 1.79 Mg ha^-1^ (5.9%) reduction in grain yield compared to the FC-80, but the difference was not significant as already noted.

Unlike grain yield, the main effects of the irrigation schedule were not significant on the plant height under both the overhead irrigation and SSDI systems. The main effects of nutrient stress were, however, significant on the plant height under the SSDI system (**Supplementary Table S3**). The ESN-stress significantly decreased plant height compared to the NN-stress under both FC-50 (by 9.38%) and FC-80 (by 6.43%) (**Figures 2C, D**). Under the overhead irrigation, the FC-80 also resulted in increased plant height compared to the FC-50, albeit the differences were not significant. The main effects of nutrient stress were significant on the stover under both the overhead irrigation and SSDI systems, but the main effects of the irrigation schedule were significant under just the SSDI system (**Supplementary Table S3**). Compared to the NN-stress, the ESN-stress decreased the stover by 29.9% (FC-50) and 21.9% (FC-80) under the overhead irrigation and by 34.6% (FC-50) and 31.6% (FC-80) under the SSDI (**Figures 2E, F**).

In contrast, the ESN-stress increased the harvest index over the NN-stress under both the overhead irrigation and SSDI systems (**Supplementary Table S3** and **Figures 2G, H**). Although the differences were statistically significant, the magnitude was quite marginal, with the ESN-stress increasing harvest index over the NN-stress by just 3.3% (FC-50) and 2.8% (FC-80) under the overhead irrigation and by 3.1% (FC-50) and 3.9% (FC-80) under the SSDI (**Figures 2E, F**). The main effects of the irrigation schedule, and the interaction effects of the irrigation schedule and nutrient stress, were not significant on the TSW under both the overhead irrigation and SSDI systems (**Supplementary Table S3** and **Figures 2I, J**). However, the main effects of nutrient stress were significant on the TSW under the SSDI. The ESN-stress decreased the TSW by 7.77% under the FC-50 and by 4.80% under the FC-80 compared to the NN-stress.

As already noted, yield reduction from the early season nutrient stress under the SSDI system was fairly high. The results could be due to the dry conditions of the topsoil layer under the SSDI field, evident by the soil moisture sensors (Akıncı and Lösel, 2012; Bárzana and Carvajal, 2020; Sah et al., 2020). The soil moisture sensors at the 10-cm depth of the SSDI field showed the water content was below the permanent wilting point for a prolonged period. Reduced nutrient uptake due to drought stress can result in a reduction in cell expansion, stunted growth, and increased susceptibility to pests and diseases, all of which will eventually lead to reduced biomass, yield, stover, and TSW as observed in this study (Hussain et al., 2019; Bárzana and Carvajal, 2020). The inability of corn to fully recover from the early season nutrient stress highlights the sensitivity of corn in the early season. Studies show that stress conditions cause corn to transition through its developmental stages quickly (Silva and Uchida, 2000; Grant et al., 2001; Roth et al., 2023). The ESN-stress, however, resulted in a higher harvest index than the NN-stress. Harvest index is a measure of the efficiency of plant biomass partitioned to the grain (Smith et al., 2018; Porker et al., 2020). Prevailing environmental conditions can cause an increase or decrease in the harvest index of corn. Generally, crops survive stress by reducing the length of the vegetative stage, which limits the growth of the vegetative parts to partition photo-assimilates to the seeds (Unkovich et al., 2010; Cohen et al., 2021).

### 3.6 Ear development

**Figure 3:**
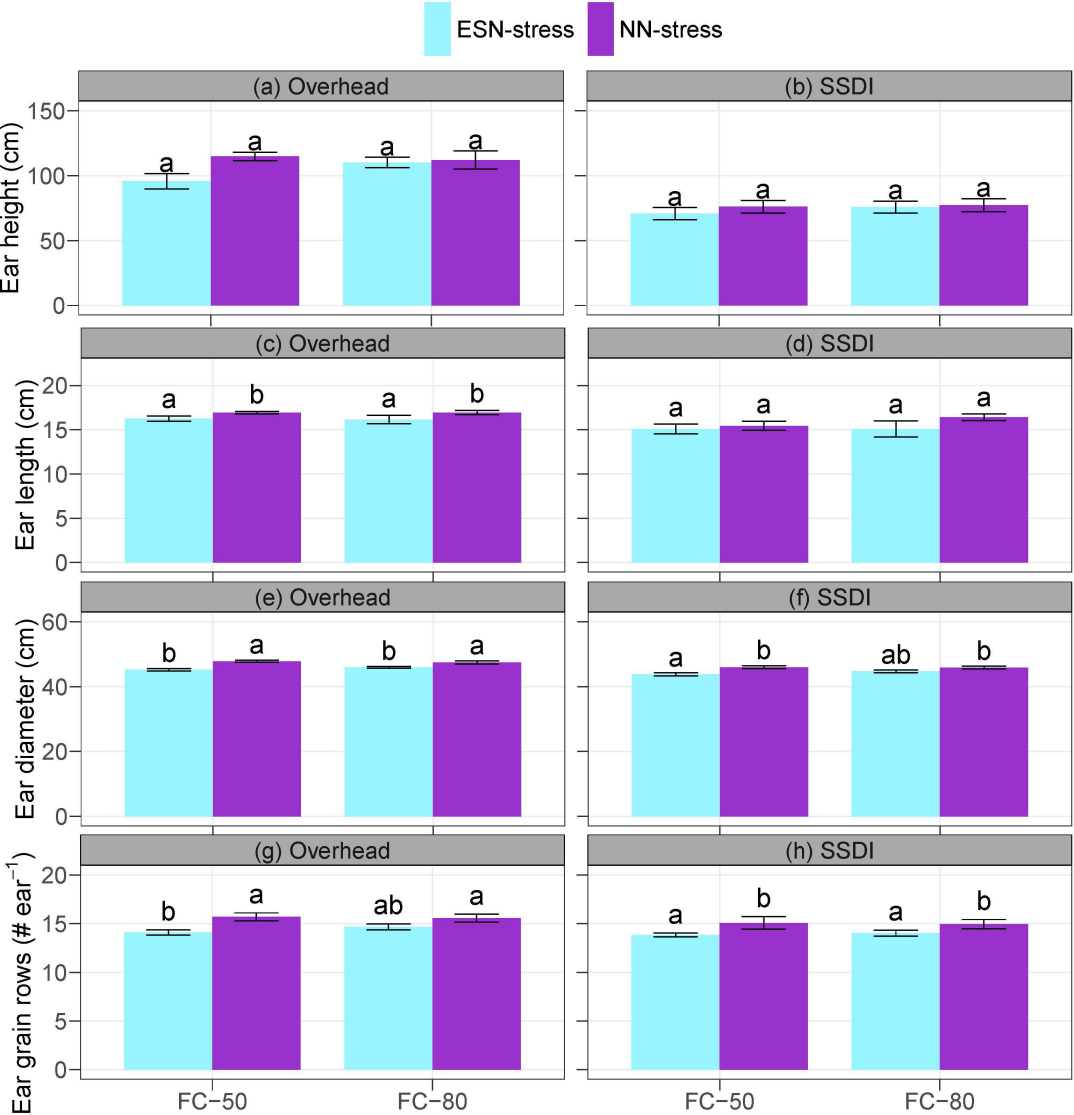
Effects of irrigation schedule and nutrient stress on corn ear development **(A–H)** under overhead irrigation and SSDI systems. Within the irrigation application method (overhead or SSDI) and measurement variable, bar plots of means not sharing any letter are significantly different using the least squares means and adjusted Tukey multiple comparisons (*P* < 0.05). Error bars indicate standard errors of the mean (n = 4). SSDI, Subsurface drip irrigation; FC-50, Irrigation triggered at 50% field capacity; FC-80, Irrigation triggered at 80% field capacity; ESN-stress, early season nutrient stress; NN-stress, reduced nutrient stress.

The main effects of irrigation schedule and the interaction effects of irrigation schedule and nutrient stress were not significant on the ear height, ear length, ear diameter, and grain rows. However, the main effects of nutrient stress had significant impacts on ear length, ear diameter, and ear grain rows under the overhead irrigation and on the ear diameter and ear grain rows under the SSDI (**Supplementary Table S3**). Compared to the NN-stress, the ESN-stress resulted in a general reduction in all the measured ear development parameters, ranging from 1.7% to 20% reduction in ear height, 2.4% to 8.7% reduction in ear length, 6.2% to 11.4% reduction in ear grain rows, and 2.6% to 5.9% reduction in ear diameter across the overhead irrigation and SSDI systems (**Figure 3**). The results indicate the early season nutrient stress affected the source and sink dynamics. The use of nutrients entails uptake, assimilation, translocation, and remobilization during senescence (Masclaux-Daubresse et al., 2010; Sintim et al., 2015, 2016). As already mentioned, nutrient stress can cause the plant to shorten the vegetative stage and progress to the reproductive stage (Silva and Uchida, 2000; Grant et al., 2001; Roth et al., 2023). Thus, plants may not accumulate adequate nutrients at the vegetative stage to translocate and remobilize, which could subsequently impact the ear development of corn, as observed in the ESN-stress plots (Unkovich et al., 2010; Cohen et al., 2021).

## 4 Conclusions

Inducing early season nutrient stress in corn by delaying fertilizer application until the V6 growth stage resulted in biomass accumulation that was reduced by over 50% than non-stressed crops even though all plots received the same nutrient amount. Consequently, the uptake of all nutrients was significantly lower under the early season nutrient stress conditions. Soil moisture levels affected the severity of the early season nutrient stress on N accumulation, with a greater reduction in N concentration from the early season nutrient stress being more severe under the FC-50 than under the FC-80 (17.7-×4.3% vs. 12.0-17.4%). Overall, corn recovery from the early season nutrient stress was low, with the early season nutrient stress causing yield reduction of 1.58 Mg ha^-1^ (11.7%) and 1.86 Mg ha^-1^ (13.0%), respectively, for the FC-50 and FC-80 under the overhead irrigation system, and 2.95 Mg ha^-1^ (38.2%) and 3.4 Mg ha^-1^ (37.6%), respectively, for the FC-50 and FC-80 under the SSDI when compared to non-stress conditions. The findings of the study highlight that although corn has low nutrient requirement at the early growth stage, it should not be allowed to undergo nutrient stress in the early season as it will not fully recover, resulting in poor growth and reduced yield potential. Further studies are also needed to determine the optimum nutrient supply for corn at the initial growth stages.

## Data availability statement

The raw data supporting the conclusions of this article will be made available by the authors, without undue reservation.

## Author contributions

SA: Conceptualization, Data curation, Formal Analysis, Investigation, Methodology, Visualization, Writing – original draft, Writing – review and editing. GA: Data curation, Investigation, Methodology, Writing – review and editing. RL: Conceptualization, Funding acquisition, Writing – review and editing. CP: Data curation, Funding acquisition, Investigation, Methodology, Resources, Validation, Writing – review and editing. BW: Data curation, Investigation, Methodology, Resources, Writing – review and editing. WP: Conceptualization, Funding acquisition, Writing – review and editing. SV: Funding acquisition, Writing – review and editing. CB: Data curation, Writing – review and editing. GV: Funding acquisition, Writing – review and editing. GH: Supervision, Writing – review and editing. MC: Supervision, Writing – review and editing. DF: Supervision, Writing – review and editing. JD-P: Supervision, Writing – review and editing. HS: Conceptualization, Data curation, Formal Analysis, Funding acquisition, Investigation, Methodology, Project administration, Resources, Supervision, Validation, Visualization, Writing – original draft, Writing – review and editing.
