# Supplementary material for: Assessing corn recovery from early season nutrient stress under different soil moisture regimes
Source: Front Plant Sci. 2024 Mar 6;15:1344022. doi: 10.3389/fpls.2024.1344022 (PMC10950915; doi:10.3389/fpls.2024.1344022)
Supplement: Supplementary file 1 [file DataSheet_1.docx]

Supplementary Material

# Supplementary Tables

**Table S1:** *P*-values of the main effects and interaction effects of irrigation scheduling and nutrient stress on nutrient concentrations of corn biomass sampled at the vegetative stage under overhead irrigation and SSDI systems.

| Effects | N | P | K | Mg | Ca | S | B | Zn | Mn | Fe | Cu | Mo |
| --- | --- | --- | --- | --- | --- | --- | --- | --- | --- | --- | --- | --- |
|  | ----------------------------------------------------------- Overhead -------------------------------------------------------- | | | | | | | | | | | |
| IS | **0.046** | 0.927 | 0.669 | 0.833 | 0.641 | 0.787 | 0.972 | 0.597 | 0.943 | 0.499 | 0.617 | 0.587 |
| NS | **0.000** | **0.001** | 0.305 | **0.003** | **0.001** | 0.393 | 0.287 | **0.000** | **0.000** | 0.088 | 0.382 | **0.001** |
| IS×NS | **0.020** | 0.243 | 0.676 | 0.386 | 0.855 | 0.882 | 0.738 | 0.520 | 0.934 | 0.706 | 0.660 | 0.277 |
|  | ------------------------------------------------------------ SSDI ------------------------------------------------------------- | | | | | | | | | | | |
| IS | **0.024** | 0.477 | 0.610 | 0.869 | 0.939 | 0.137 | 0.333 | 0.795 | 0.654 | 0.907 | 0.701 | 0.895 |
| NS | **0.000** | 0.077 | 0.232 | 0.267 | **0.029** | 0.222 | **0.000** | **0.026** | **0.013** | **0.003** | **0.006** | **0.000** |
| IS × NS | 0.590 | 0.491 | 0.611 | 0.660 | 0.604 | 0.662 | 0.153 | 0.401 | 0.709 | 0.296 | 0.913 | 0.384 |

Bold texts highlight significant effects (*P* < 0.05). SSDI: Subsurface drip irrigation; IS: Irrigation schedule; NS: Nutrient stress.

**Table S2:** *P*-values of the main effects and interaction effects of irrigation scheduling and nutrient stress on biomass and nutrient uptake of corn sampled at the vegetative stage under overhead irrigation and SSDI systems.

| Effects | Biomass | N | P | K | Mg | Ca | S | B | Zn | Mn | Fe | Cu | Mo |
| --- | --- | --- | --- | --- | --- | --- | --- | --- | --- | --- | --- | --- | --- |
|  | --------------------------------------------------------------- Overhead ------------------------------------------------------------- | | | | | | | | | | | | |
| IS | 0.051 | **0.039** | 0.140 | **0.035** | **0.032** | **0.037** | 0.059 | 0.071 | 0.063 | 0.142 | 0.121 | 0.062 | 0.051 |
| NS | **0.000** | **0.000** | **0.000** | **0.000** | **0.000** | **0.000** | **0.000** | **0.000** | **0.000** | **0.000** | **0.000** | **0.000** | **0.002** |
| IS×NS | 0.125 | **0.019** | 0.402 | 0.069 | 0.333 | 0.125 | 0.112 | 0.131 | 0.264 | 0.156 | 0.193 | 0.253 | 0.781 |
|  | ----------------------------------------------------------------- SSDI ----------------------------------------------------------------- | | | | | | | | | | | | |
| IS | 0.514 | 0.788 | 0.434 | 0.433 | 0.525 | 0.599 | 0.848 | 0.487 | 0.890 | 0.513 | 0.825 | 0.896 | 0.334 |
| NS | **0.000** | **0.000** | **0.000** | **0.000** | **0.000** | **0.000** | **0.000** | **0.000** | **0.000** | **0.000** | **0.000** | **0.000** | **0.004** |
| IS×NS | 0.963 | 0.926 | 0.929 | 0.935 | 0.821 | 0.809 | 0.870 | 0.813 | 0.775 | 0.767 | 0.913 | 0.714 | 0.570 |

Bold texts highlight significant effects (*P* < 0.05). SSDI: Subsurface drip irrigation; IS: Irrigation schedule; NS: Nutrient stress.

**Table S3:** *P*-values of the main effects and interaction effects of irrigation scheduling and nutrient stress on growth and yield parameters of corn under overhead irrigation and SSDI systems.

| Effects | Grain  yield | Plant  height | Stover | Harvest  index | TSW | Ear  height | Ear  length | Ear  diameter | Ear grain rows |
| --- | --- | --- | --- | --- | --- | --- | --- | --- | --- |
|  | -------------------------------------------------- Overhead -------------------------------------------------- | | | | | | | | |
| IS | 0.099 | 0.354 | 0.660 | 0.584 | 0.937 | 0.301 | 0.841 | 0.323 | 0.382 |
| NS | **0.003** | 0.244 | **0.000** | **0.003** | 0.145 | 0.065 | **0.023** | **0.000** | **0.000** |
| IS×NS | 0.913 | 0.298 | 0.581 | 0.909 | 0.852 | 0.120 | 0.971 | 0.063 | 0.111 |
|  | -------------------------------------------------- SSDI -------------------------------------------------- | | | | | | | | |
| IS | **0.039** | 0.337 | **0.034** | 0.795 | 0.867 | 0.445 | 0.243 | 0.426 | 0.892 |
| NS | **0.000** | **0.029** | **0.000** | **0.018** | **0.008** | 0.400 | 0.148 | **0.002** | **0.012** |
| IS×NS | 0.831 | 0.132 | 0.878 | 0.621 | 0.421 | 0.611 | 0.444 | 0.267 | 0.682 |

Bold texts highlight significant effects (*P* < 0.05). SSDI: Subsurface drip irrigation; IS: Irrigation schedule; NS: Nutrient stress; TSW: Thousand seed weight.

# Supplementary Figure


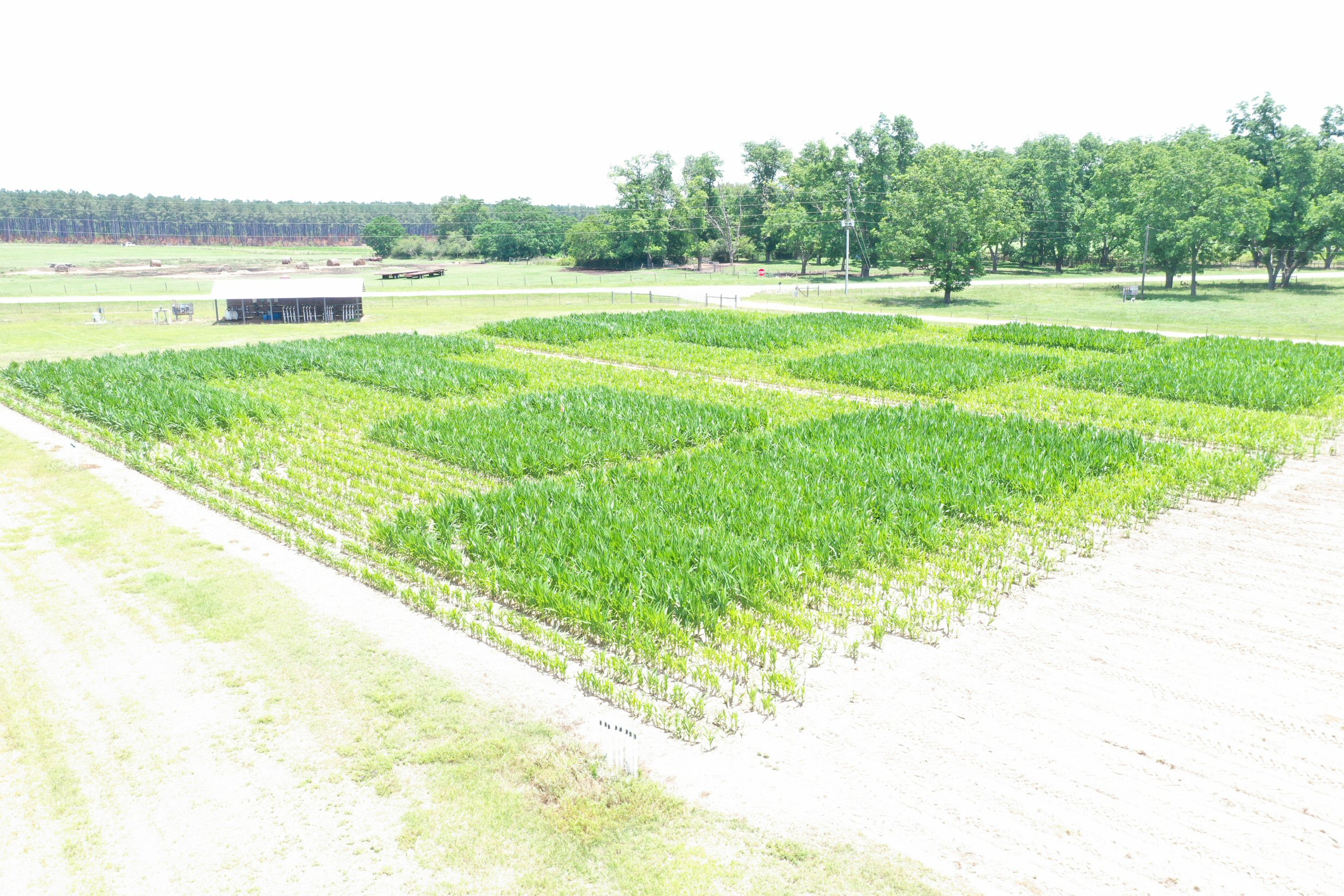


**Figure S1.** Drone image of the subsurface drip irrigation field in Camilla, GA, USA, showing poor growth of the early season nutrient stress plots. The early season nutrient stress plots received no nutrient application until the V6 growth stage.
